# Molecular Analysis of *14-3-3* Genes in *Citrus sinensis* and Their Responses to Different Stresses

**DOI:** 10.3390/ijms22020568

**Published:** 2021-01-08

**Authors:** Shiheng Lyu, Guixin Chen, Dongming Pan, Jianjun Chen, Wenqin She

**Affiliations:** 1 College of Horticulture, Fujian Agriculture and Forestry University, Fuzhou 350002, Fujian, China; 2140305002@fafu.edu.cn (S.L.); 1170371005@fafu.edu.cn (G.C.); pdm666@fafu.edu.cn (D.P.); 2 Mid-Florida Research and Education Center, Department of Environmental Horticulture, Institute of Food and Agricultural Sciences, University of Florida, Apopka, FL 32703, USA

**Keywords:** abiotic stress, CitGF14s, citrus canker, *Citrus sinensis*, sweet orange, 14-3-3s

## Abstract

14-3-3 proteins (14-3-3s) are among the most important phosphorylated molecules playing crucial roles in regulating plant development and defense responses to environmental constraints. No report thus far has documented the gene family of *14-3-3s* in *Citrus sinensis* and their roles in response to stresses. In this study, nine *14-3-3* genes, designated as *CitGF14s* (*CitGF14a* through *CitGF14i*) were identified from the latest *C. sinensis* genome. Phylogenetic analysis classified them into ε-like and non-ε groups, which were supported by gene structure analysis. The nine *CitGF14s* were located on five chromosomes, and none had duplication. Publicly available RNA-Seq raw data and microarray databases were mined for 14-3-3 expression profiles in different organs of citrus and in response to biotic and abiotic stresses. RT-qPCR was used for further examining spatial expression patterns of *CitGF14s* in citrus and their temporal expressions in one-year-old *C. sinensis* “Xuegan” plants after being exposed to different biotic and abiotic stresses. The nine *CitGF14s* were expressed in eight different organs with some isoforms displayed tissue-specific expression patterns. Six of the *CitGF14s* positively responded to citrus canker infection (*Xanthomonas axonopodis* pv. *citri*). The *CitGF14s* showed expressional divergence after phytohormone application and abiotic stress treatments, suggesting that 14-3-3 proteins are ubiquitous regulators in *C. sinensis*. Using the yeast two-hybrid assay, CitGF14a, b, c, d, g, and h were found to interact with CitGF14i proteins to form a heterodimer, while CitGF14i interacted with itself to form a homodimer. Further analysis of *CitGF14s* co-expression and potential interactors established a 14-3-3s protein interaction network. The established network identified *14-3-3* genes and several candidate clients which may play an important role in developmental regulation and stress responses in this important fruit crop. This is the first study of 14-3-3s in citrus, and the established network may help further investigation of the roles of 14-3-3s in response to abiotic and biotic constraints.

## 1. Introduction

Plants are constantly exposed to different abiotic and biotic stresses, including drought, extreme temperatures, high salinity, and various pathogens. Due to their sessile nature, plants have evolved a series of mechanisms to cope with the environmental challenges. Plant 14-3-3s, encoded by genes called general regulatory factors [1,2], regulate critical biochemical processes and sophisticated signaling networks in plants though protein–protein interactions by binding to phosphorylated protein clients [3]. 14-3-3 proteins were originally isolated from mammalian brain tissue and were named according to their elution and migration pattern on DEAD-cellulose chromatography and starch-gel electrophoresis [4]. The 14-3-3s are highly conserved proteins and exist in all eukaryotes with multiple isoforms. Yeast has two genes encoding 14-3-3s [5], animals typically have seven [6], and plants have more *14-3-3* genes: 13 in *Arabidopsis* [6], 17 in tobacco (*Nicotiana tabacum*) [7], 12 in tomato (*Solanum lycopersicum*) [8], and eight in rice (*Oryza sativa*) [9].

The protein sequence of 14-3-3s can be divided into three regions: A variable N-terminus, a conserved core region, and a variable C-terminus. Based on the gene structure, plant 14-3-3 proteins are divided into two distinct groups, namely epsilon (ε) and non-epsilon, and the latter is plant-specific [2,10,11]. Crystal structure studies show that 14-3-3 dimers consist of a typical clamp shape structure containing alpha helical amphipathic grooves formed by a monomer [12,13]. The monomer can interact with phosphorylated proteins; thus, the groove is the main target binding site [14]. Large-scale interactomics and mass-spectrometry-based studies have identified more than 300 potential 14-3-3 targets in plant [3]. There are about a dozen 14-3-3 proteins forming homodimers and heterodimers that function to reverse phosphorylation of proteins in plants [15,16]. Three canonical phosphorylation-dependent 14-3-3 binding motifs can be recognized by all isoforms: RSXpSXP (mode-I), RXXXpSXP (mode-II), and pS/pTX1–2-COOH (mode-III) (where R, S, and P represents arginine, serine, and proline, X is any amino acid, pS is phosphoserine) [12,17,18]. Phosphorylation is an essential posttranslational modification, which is fast and reversible and affects thousands of proteins and regulates a plethora of different processes in plants [19]. Protein phosphorylation occurs mainly on serine (pS), threonine (pT), and tyrosine (pY) residues. The 14-3-3 proteins can also bind non-phosphorylated targets, such as WLDLE [20] and GHSL [20,21].

A growing body of evidence indicates that the 14-3-3s can regulate plant responses to abiotic and biotic stresses [16,19,22,23,24]. 14-3-3s play important roles in plant tolerance to salinity and drought. *Arabidopsis* 14-3-3s κ and λ have been reported to inhibit the SOS (salt overly sensitive) pathway by repressing SOS2 kinase activity in the absence of salt stress [25]. Rice 14-3-3 family genes were named as *GF14a* through *GF14h*, and four members (*GF14b*, *GF14c*, *GF14e*, and *GF14f*) were all induced by PEG6000 (drought-mimic) treatments [26]. The 14-3-3s have been reported to regulate plant cold tolerance. RARE COLD INDUCIBLE 1A (RCI1A) and RCI1B were the first two 14-3-3 proteins that were demonstrated to be induced by cold stress in *Arabidopsis* [27]. The kinetics of RCI1A and RCI1B mRNA accumulation induced by cold stress is correlated with the increased freezing tolerance that occurs during the cold acclimation process in *Arabidopsis*, implying that these genes play pivotal roles in this adaptive process [27,28]. The 14-3-3 proteins are also involved in regulation of nutrient stress, such as low phosphorus stress [29], iron deficiency [30], wounding [28], and ABA signal [31]. 14-3-3 proteins respond to pathogen infection by changing transcript levels or, in some instances, protein abundance or properties, or both [19]. Tomato *TFT1*, *TFT4*, and *TFT6* genes were upregulated in the Cf-9-mediated hypersensitive response (HR) [32]. Similarly, *14-3-3* genes are expressed during a race-specific HR of soybean inoculated with *Pseudomonas syringae* [33] and upon a resistant reaction to the soybean cyst nematode [34]. Tobacco 14-3-3 isoform h is induced after inoculation with tobacco mosaic virus (TMV) [7]. A *Gossypium hirsutum 14-3-3* is rapidly expressed in response to *Verticillium dahliae* [35] in a cultivar with enhanced wilt resistance, which may suggest a specific role for *14-3-3* in resistance to the pathogen.

Citrus fruits are among the highest value fruit crops in terms of nutritional components and international trade. Citrus crop production constantly encounters both abiotic and biotic stresses, such as drought, salinity, cold, and pathogens, which have significantly affected citrus production worldwide. A better understanding of citrus responses to these constraints will improve breeding strategies and production practices for increased resistance or tolerance to stresses. Plant *14-3-3s* as general regulatory factors may play important roles in citrus responses to these stresses. The genome-wide analysis of *14-3-3* family genes has been identified from various plants, including *Arabidopsis* [6], soybean (*Glycine max*) [36], common bean (*Phaseolus vulgaris*) [37], rice [26], black cottonwood (*Populus trichocarpa*) [38], and foxtail millet (*Setaria italica*) [39]. Up to now, there have been no reports on *14-3-3* family genes in citrus. 

In this study, we report a comprehensive genomic identification and phylogenetic analysis of nine members of the *14-3-3* gene family in sweet orange (*Citrus sinensis*) and document their expression profiles in different organs and their responses to abiotic and biotic stresses as well as hormone signal. Our results for the first time provide fundamental information about *14-3-3* genes and their responses to stresses in this citrus species.

## 2. Results

### 2.1. 14-3-3 Identification, Phylogenetic Analysis, and Function Prediction

A total of 13 putative *14-3-3* genes were identified from the whole genome of *C. sinensis* using the *14-3-3* genes from *Arabidopsis*, soybean, and black cottonwood as queries. After removing incomplete and redundant sequences, nine *14-3-3* genes were confirmed. They were designated as *CitGF14* (*CitGF14a* through *CitGF14i*) (Table 1). Their open reading frames ranged from 741 to 798 bp encoding 247 to 266 amino acids with putative MW varying from 27.9 to 30.2 kDa. The theoretical isoelectric points ranged from 4.69 to 5.14. Corresponding proteins were predicted to localize in the cytoplasm (cyto), chloroplast (chlo), nucleus plasma (nucl plas), and plasma membrane (plas) depending on individual proteins (Table 1)

**Figure 1:**
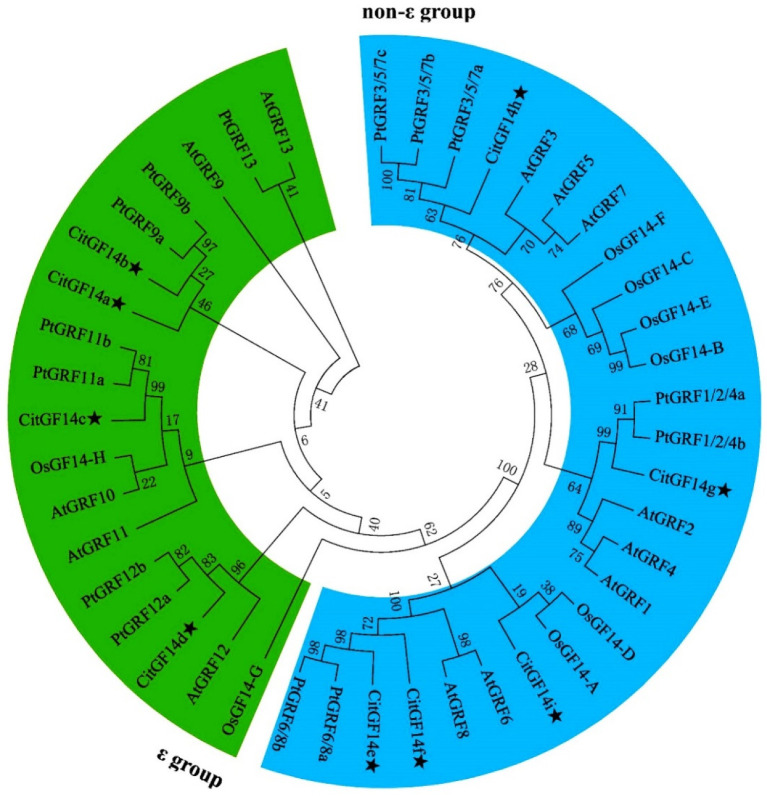
Phylogenetic trees of *14-3-3* family genes in *Citrus sinensis* (*CitGF14s*, indicated by the star symbol), *Arabidopsis* (*AtGRF*), *Populus *trichocarpa (PtGRF)**, and *Oryza sativa* (*OsGRF*) constructed using the MEGA6.0 program with neighbor-joining method within 1000 bootstrap replicates. The green and blue shade separates the ε and non-ε groups.

The evolutionary relationships of 44 *14-3-3s* from *Arabidopsis*, rice, black cottonwood, and *C. sinensis* were phylogenetically analyzed. Eighteen of them were clustered into ε-like groups, and 26 were clustered into non-ε groups (Figure 1). Four *CitGF14s* (*CitGF14a*, *b*, *c*, and *d*) were grouped into ε-like isoforms, and the remaining five *CitGF14s* were grouped into non-ε isoforms. None of the *CitGF14s* were duplicated.

### 2.2. Localization in Chromosomes and Gene Structure

**Figure 2:**
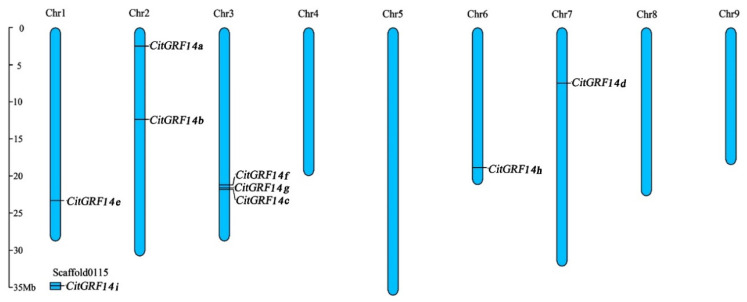
Genomic distribution of *14-3-3* (*CitGF14s*) genes across nine *Citrus sinensis* chromosomes. The chromosome number is indicated at the top of each chromosome. The scale is in megabases (Mb). Chromosomal locations of *CitGF14s* were indicated based on the physical position of each gene.

Eight *14-3-3* genes (*CitGF14a* through *CitGF14h*) were located on five chromosomes (Chr 1, 2, 3, 6, and 7) of *C. sinensis* (Figure 2), yet *CitGF14i* could not be mapped on a chromosome and remained as unanchored scaffolds. Both *CitGF14a* and *CitGF14b* were situated on chromosome 2; *CitGF14e*, *CitGF14h*, and *CitGF14d* were located on chromosomes 1, 6, and 7, respectively; while *CitGF14c*, *CitGF14f*, and *CitGF14g* were linked on chromosome 3.

**Figure 3:**
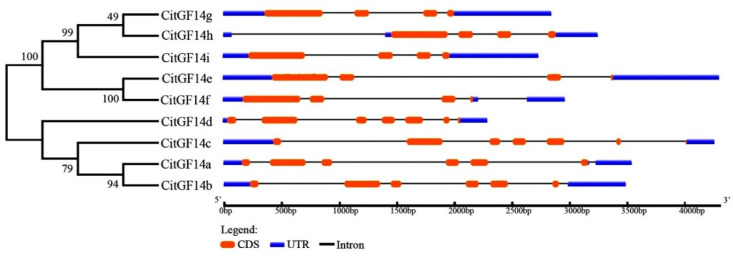
Structure analysis of *CitGF14s* genes in *Citrus sinensis*. The graphic representation is displayed using GSDS (http://gsds.cbi.pku.edu.cn/). The unrooted phylogenetic tree was constructed using the amino sequences of *CitGF14s* genes by the Neighbor-Joining method with 1000 bootstrap replicates. Exons consisted of CDS shown as orange boxes, introns are shown as thin lines, and UTRs are shown as blue boxes.

Gene structure analysis showed that *CitGR14s* contained 3 to 6 exons, interspersed by highly distinct introns (Figure 3). Four ε-like *CitGF14s* showed six conserved exons interrupted by intron and UTR in different lengths. Non-ε *CitGF14* genes were also interrupted by introns. *CitGF14e*, *CitGF14g*, and *CitGF14i* carried three introns, *CitGF14f* and *CitGF14h* possessed four introns, the remaining had six introns.

### 2.3. CitGF14s Sequence Alignment

Amino acid sequence alignment showed that the deduced *CitGF14s* from *C. sinensis* were highly conserved with the exception of the N-terminal and C-terminal regions. Nine α-helices were shown in green rectangular boxes (Additional file 1: Figure S1A). Two conserved signature motifs RNL(L/V)SV(G/A)YKNV and YKDSTLIMQLLRDNLTLWTS were found in α-helix 3 and α-helix 9, respectively.

Protein CitGF14b, CitGF14d, CitGF14e, CitGF14f, CitGF14g, CitGF14h, and CitGF14i had rather similar three-dimensional structures based on the Swiss-model prediction (Additional file 1: Figure S1B). All these similar proteins contained two potassium channel KAT1 ligands except for CitGF14b. The remaining two proteins (CitGF14a and CitGF14c) had relatively simple structures with no ligands.

### 2.4. Cis-Regulatory Elements

Different cis-acting elements related to plant growth, development, and stress responses were identified in *CitGR14s* (Additional file 2: Table S1). The circadian element was presented in all *CitGF14* promoters except for *CitGF14d*. The Skn-1 motif required for endosperm expression was highly conserved in six *CitGF14* promoters. The ABA responsive element ABRE was presented in *CitGF14b*, *CitGF14c*, *CitGF14e*, and *CitGF14g*. Box-W1, a fungal elicitor responsive element, was a highly conserved stress-related element which was found in five of nine *CitGF14s*. The upstream flanking regions of *CitGF14g* contained 11 stress-responsive promoters, including ABRE, ARE, CGTCA-motif, ERE, GC-motif, HSE, MBS, P-box, TC-rich repeats, TCA-element, TGA-element, and GACG-motif. Moreover, other cis-acting elements associated with biotic and abiotic stress responses, such as WUN-motif, GARE-motif, AuxRR-core, and SARE were also identified.

### 2.5. Tissue-Specific Expression Patterns of CitGF14s

**Figure 4:**
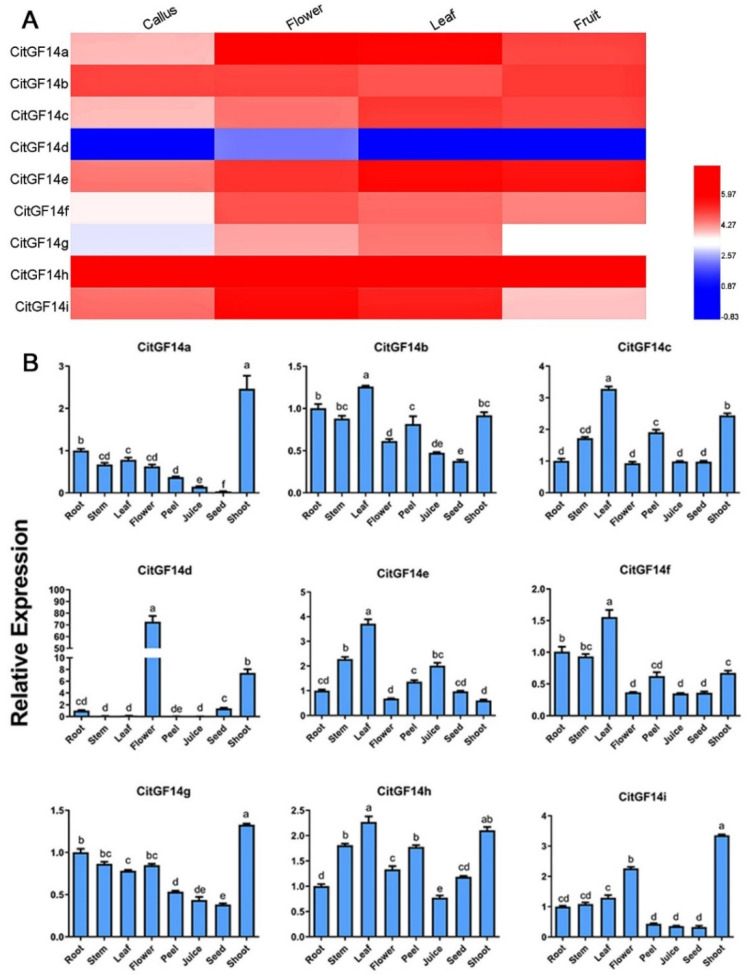
Expression analysis of *CitGF14s* in different tissue and organs of *Citrus sinensis* plants. (**A**) Expression profiles of *CitGF14s* derived from RNA-Seq data where RPKM expression values were log-transformed for normalization. Log2 based RPKM values were used for creating the heat map with clustering by HemI. The scale represents the relative intensity of RPKM values. (**B**) RT-qPCR analysis of the expression patterns of *CitGF14s* in different organs of *C. sinensis*. The relative expression was normalized using the ACTIN and GAPDH genes as references. Each bar represented the mean of four biological replications with standard error. Different letters on the top of bars indicate significant differences analyzed by Tukey’s HSD test at *p* < 0.05 level.

The expression of *CitGF14s* in callus, leaf, flower, and fruit, which were mined from the RNA-Seq raw data of *C. sinensis* genome database (http://citrus.hzau.edu.cn/orange/) is presented in Figure 4A. *CitGF14a* and *CitGF14i* were strongly expressed in flowers and leaves. The expression of *CitGF14e* in flower was higher than in callus and then further increased in leaves and fruit. *CitGF14b* expression was induced in callus and flower, slightly decreased in leaves, and then increased in fruit. *CitGF14h* was highly induced and constantly expressed in all four tissues or organs. On the other hand, *CitGF14d* was down regulated in callus, leaves, and fruit. Other *CitGF14s* were either slightly or moderately induced depending on tissue or organ.

The spatial expressions of *CitGF14s* were further analyzed by RT-qPCR in *C. sinensis* “Xuegan” when plants were not exposed to any stresses (Figure 4B). *CitGF14s* were not highly induced in roots and stems except for *CitGF14e* and *CitGF14h* in stems that had over a 1.5-fold increase. The expression of *CitGF14a*, *c*, *d*, *h*, and *i* in shoots and *CitGF14c*, *e*, and *h* in leaves was highly induced, which was largely similar to those mentioned in above RNA-Seq data with the exception of *CitGF14d* that was primarily down regulated in callus, leaves, and fruit based on the RNA-Seq data (Figure 4A), but it was highly upregulated in flower as well as shoots in the RT-qPCR analysis. Furthermore, the expression of all *CitGF14s* was low in peel, juice, and seeds. 

### 2.6. Responses to Infection of Citrus Canker and Citrus Greening Pathogens

**Figure 5:**
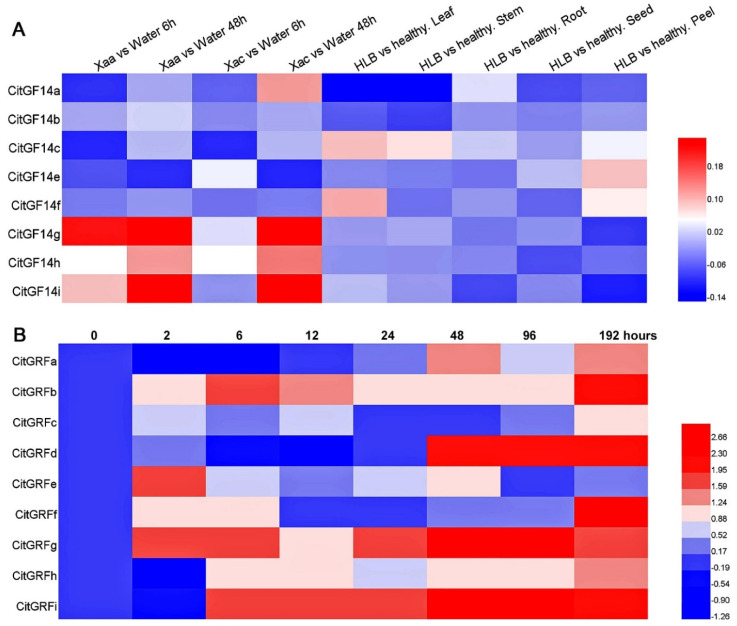
Expression profiles of *CitGF14s* in response to different stresses. (**A**) The microarray data was downloaded from NCBI database. Heatmap shows hierarchical clustering of *CitGF14* expression after plants were inoculated with citrus canker pathogens: *Xanthomonas axonopodis* pv. *citri* (Xaa) and *Xanthomonas axonopodis* pv. *Aurantifolii* (Xac) and citrus greening pathogen: *Candidatus* Liberibacter asiaticus (*Ca*. Las). Gene expression values were calculated based on the ratios between the infection and the mock (control). Heatmap was generated based on log2 (infection expression/mock expression) values by using HemI. The color scale represents the relative intensity level of transcript abundance. (**B**) RT-qPCR analysis of *CitGF14s* expression after *C. sinensis* “Xuegan” was inoculated with Xac. The relative expression was normalized using the ACTIN and GAPDH genes as references using 2^−ΔΔ^Ct method. The values were based on the means of four biological replications.

Affymetrix microarrays data were mined in this study for potential roles of *CitGF14* genes in response to citrus canker. Results showed that all citrus probe sets contained less than 10,000 genes, indicating the not all citrus *CitGF14s* had been covered by the microarray data. For example, the probe signal for *CitGF14d* was not detected in the microarray. Based on eight *CitGF14s* from the microarray data, their responses to citrus canker infection are presented in Figure 5A (first four columns). *CitGF14g* was induced by *Xanthomonas axonopodis* pv. *citri* (Xaa) 6 to 48 h after infection and by *Xanthomonas axonopodis* pv. *Aurantifolii* (Xac) 48 h after infection (Figure 5A). The inoculation of Xaa and Xac respectively induced *CitGF14i* expression only 48 h after infection. *CitGF14h* was slightly induced by Xaa and Xac 48 h after infection. The other *CitGF14s* did not respond to the infection of the two pathogens. 

The infection of Xac to *C. sinensis* “Xuegan” caused downregulation of *CitGF14a* in 2 to 6 h and then variable expression thereafter till 192 h (Figure 5B). *CitGF14b* was induced 6 h after inoculation, and its expression was then reduced from 12 to 96 h but highly increased at 192 h. *CitGF14d* was highly induced from 48 h to 192 h. *CitGF14g* responded quickly, 2 h after the inoculation, and reached the highest expression level from 48 h to 96 h. There was a downregulation of *CitGF14i* initially, its expression increased from 6 h to 24 h, and attained the highest expression level from 48 h to 192 h. The active responses of *CitGF14g* and *CitGF14i* largely concurred with the above microarray results. The other *CitGF14s* showed varied levels of down or upregulation over the 192-h evaluation period. 

Microarray data were also explored for potential roles of *CitGF14s* in response to the infection of citrus greening: *Candidatus* Liberibacter asiaticus (*Ca.* Las) (Figure 5A, five columns from the right). Compared to healthy organs, there was slight increase in the expressions of *CitGF14c* and *CitGF14f* in leaves and *CitGF14e* in peel. The other genes showed little response to the infection except for *CitGF14a* that was down regulated in leaves and stem and *CitGF14g* and *CitGF14i* that were down regulated in peel. 

### 2.7. Responses to Plant Hormone Treatments

**Figure 6:**
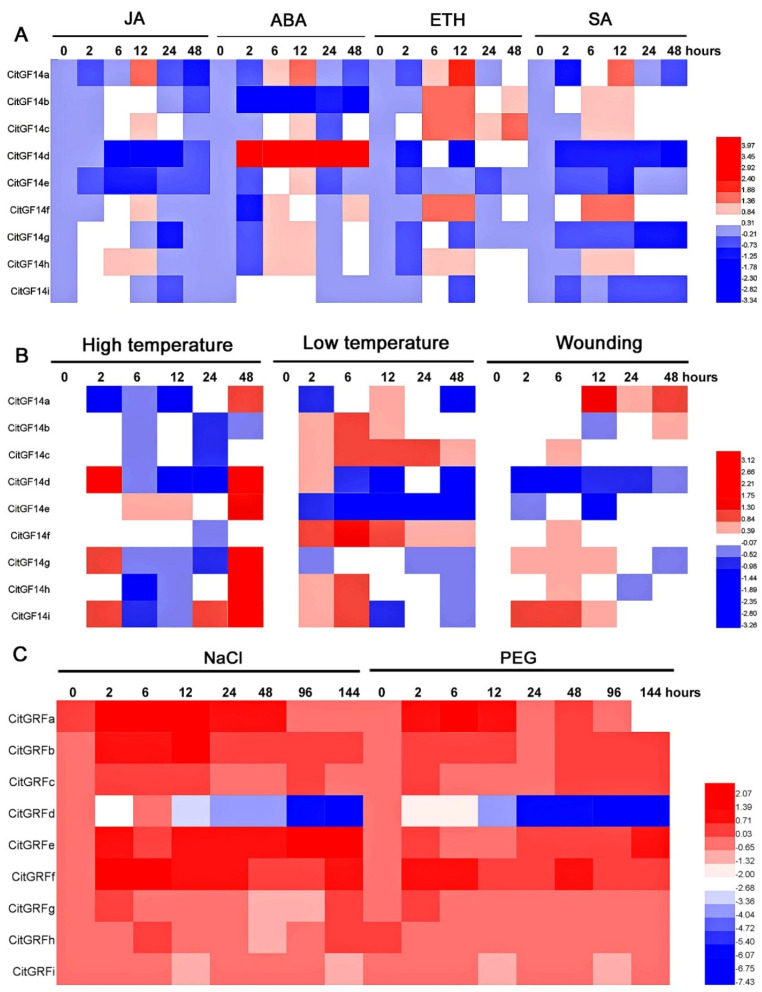
RT-qPCR analysis of *CitGF14* expressions after *C. sinensis* “Xuegan” plants were sprayed with jasmonate (JA), abscisic acid (ABA), ethephon (ETH), and salicylic acid (SA) (**A**), exposed to low (4 °C) and high (42 °C) temperatures as well as wounding (**B**), and treated with NaCl (200 mM) and polyethylene glycol (PEG) (20% PEG6000), a simulated drought stress (**C**). The relative expression was normalized using the ACTIN and F-box gene as references by 2^−ΔΔ^Ct method. Heatmaps were generated based on log2 (treatment expression/control expression) with HemI. The values were based on the means of four biological replications.

Foliar application of jasmonate (JA) on “Xuegan” caused more downregulation of *CitGF14* genes than upregulation (Figure 6A). The upregulation only occurred with *CitGF14a*, *c*, and *f* 12 h after JA application and *CitGF14h* from 6 h to 12 h. Genes with the most pronounced down regulation were *CitGF14a* at 2 h and 48 h; *CitGF14d* from 6 h to 24 h; *CitGF14e* from 6 h to 12 h; and *CitGF14g* and *i* at 24 h. *CitGF14b* and *CitGF14d* showed noticeable responses to ABA (Figure 6A). The former was primarily down regulated from 2 h to 48 h after application, whereas the latter was completely upregulated from 2 h to 48 h after application. ABA application also induced the expression of all the other *CitGF14s* at different time periods except for *CitGF14i* that was largely down regulated.

The application of ethephon (ETH) induced variable expressions of *CitGF14a*, *b*, *c*, *f*, and *h* (Figure 6A). These genes were upregulated from 6 h to 12 h after application with exception of *CitGF14b* and *c* whose expression reduced at 24 h but increased at 48 h. The other genes were primarily down regulated after ETH application. Foliar spraying of salicylic acid (SA) also induced variable expressions of *CitGF14a*, *b*, *c*, *f*, and *h* (Figure 6A). *CitGF14a* was upregulated after 12 h of application, and *CitGF14b*, *c*, *f*, and *h* were induced from 6 h to 12 h. The other *CitGF14s* were downregulated or remained unchanged. 

### 2.8. Responses to Low and High Temperatures and Wounding

The exposure of *C. sinensis* “Xuegan” plants to 42 °C led to the upregulations of five *CitGF14* genes at different times during the treatment (Figure 6B). *CitGF14a*, *e*, and *h* were either uninduced or downregulated from 0 h to 24 h but highly induced at 48 h. On the other hand, the expression levels of *CitGF14d* and *g* were higher at 2 h, decreased thereafter, and then highly increased at 48 h. *CitGF14i* had similar expression pattern as *CitGF14d* and *g*, but its increase started 24 h and sustained to 48 h. The other *CitGF14s* were either downregulated or showed little change.

Chilling treatment of “Xuegan” resulted in *CitGF14* responses opposite to the high temperature treatment. *CitGF14b*, *c*, and *f*, which were not upregulated at high temperature were strongly induced (Figure 6B). *CitGF14b* was induced at 2 h, and its expression reached the highest at 6 h, and then decreased at 12 h. *CitGF14c* was induced at 2 h, attained the highest from 6 h to 24, but decreased at 48 h. The expression of *CitGF14f* was high at 2 h and peaked at 6 h, and then gradually reduced. Additionally, *CitGF14h* and *CitGF14i* were also highly induced at 6 h. The expression of the other *CitGF14s* were variable, and largely downregulated.

Wounding of “Xuegan” plants induced the upregulation of *CitGF14a* at 12 to 48 h and *CitGF14i* from 2 h to 6 h (Figure 6B). Wounding treatment also moderately induced the expression of *CitGF14b* at 48 h and *CitGF14c*, *f*, and *h* at 6 h as well as *CitGF14g* from 2 h to 12 h, and *CitGF14i* at 12 h. The other *CitGF14s* either remained unchanged or downregulated.

### 2.9. Responses to Salinity and Drought Stresses

All *CitGF14* genes responded to the salt treatment (Figure 6C). *CitGF14a* was highly induced from 0 h to 48 h. The expressions of *CitGF14b*, *e*, and *f* were higher starting from 2 h to 144 h. The other *CitGF14s* were upregulated varying from moderate to high except for *CitGF14d* that fluctuated up and down and then remained downregulation from 24 h to 144 h. 

The simulated drought stress by polyethylene glycol (PEG) treatment showed a rather similar expression pattern of *CitGF14s* as NaCl treatment (Figure 6C). All genes were upregulated except for *CitGF14d* that was initially upregulated, then slightly decreased, and finally downregulated from 24 h to 144 h. *CitGF14a* and *b* were highly induced from 2 h to 12 h, and both became moderately induced in 24 h. The expression of *CitGF14a* was higher again at 48 h and then decreased thereafter, but *CitGF14b* maintained a high expression level from 48 h to 144 h. *CitGF14c* and *e* were highly upregulated at 2 h, remained moderate in expression from 6 h to 24 h and 6 h to 12 h, respectively, and finally highly induced thereafter. *CitGF14f* was consistently highly expressed from 2 h to 144 h. 

### 2.10. Interactions among CitGFs

**Figure 7:**
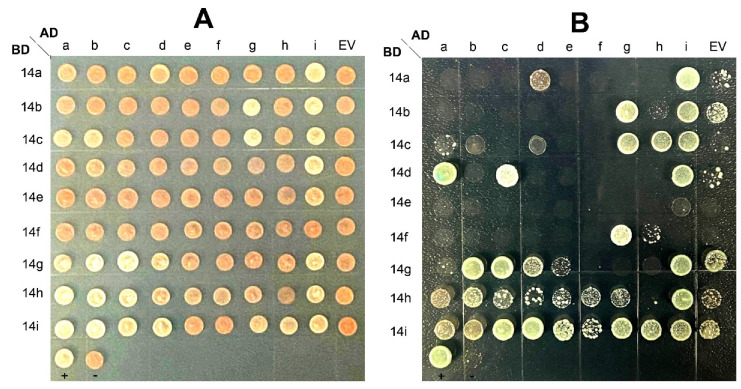
Yeast two-hybrid (Y2H) assay to test interactions among nine CitGF14 (14-3-3) proteins of *Citrus sinensis*. The coding sequences of the *CitGF14s* genes were cloned into the Y2H vectors pGADT7 (AD) and pGBKT7 (BD), and introduced into yeast cells Y2H gold. Transformants were assayed for growth on SD/-Trp-Leu (**A**) and SD/-Trp-Leu-His-Ade-x-α-gal (**B**) nutritional selection media. EV was empty vector pGADT7 used as a negative control.

To determine whether 14-3-3 proteins could interact with each other or with other proteins, we systematically assessed the interactions among all the 14-3-3 proteins using yeast two hybrid (Y2H) assay. GAL4 DNA-binding domain (BD) was fused to prey protein (CitGF14a through CitGF14i), GAL4 transcriptional activating domain (AD) was fused to bait protein (CitGF14a through CitGF14i and pGADT7 empty vector), and they were co-transformed into yeast cells. As shown in Figure 7, CitGF14a, b, c, d, g, and h interacted with CitGF14i proteins to form a heterodimer while CitGF14i interacted with itself to form a homodimer in yeast. Interactions were also found in other 14-3-3 protein pairs, CitGF14g interacted with b and c as prey or bait. CitGF14d showed strong interaction with a and c as prey, while when CitGF14*d* was bait, the interaction became weak. CitGF14h interacted with CitGF14c as bait protein, but CitGF14*h* weakly interacted with all target and empty vector. Thus, CitGF14h showed auto-activation as prey vector. 

## 3. Discussion

Plant 14-3-3 proteins play important roles in regulation of plant responses to abiotic and biotic stresses. Different number of 14-3-3 proteins have been identified and analyzed in a number of land plant species, but such information is not available in citrus. The present study for the first time documented 14-3-3 proteins in *C. sinensis* and evaluated *14-3-3* genes in response to various stresses.

### 3.1. Nine 14-3-3 Genes and Their Characteristics in C. sinensis

A total of nine *CitGF14* genes were identified in *C. sinensis* by a genome-wide search based on conserved domains and sequence similarities from known *14-3-3s*. They were divided into ε-like and non-ε groups (Figure 1), and eight of the nine were located on five chromosomes (Figure 2). Phylogenetic results were consistent with the clustering of 14-3-3 proteins in *Arabidopsis* [6], rice [40], and wheat [41]. The ε group, also known as “living fossil” 14-3-3 isoforms, is considered essential to eukaryotic biology, while the proteins in the non-ε group generally play organism-specific regulatory roles [42]. Gene structure analysis also supported this classification (Figure 3). Each of ε group genes (*CitGF14a* through *CitGF14d*) has six exons, while each of non-ε group genes (*CitGF14e* through *CitGF14i*) has three exons.

The relatively small number of *CitGF14* genes along with no duplicated ones were not surprising because there was no recent whole-genome duplication in *C. sinensis* evolution except γ event which was shared by all core eudicots [43]. Phylogentic analysis suggest that *C. sinensis 14-3-3s* are evolutionally close to *Populus* since they belonged to the same clade and they are all ancient. Amino acid sequence alignment indicated that all *C. sinensis 14-3-3s* showed a high level of amino-acid similarity except for the N-terminal and C-terminal regions (Additional file 1: Figure S1), which are similar to all other 14-3-3s [44]. These highly conserved 30-kDa acidic proteins are, each composed of approximately 250 amino acids, nine α-helices, and two conserved signature motifs RNL(L/V)SV(G/A)YKNV and YKDSTLIMQLLRDNLTLWTS [14,44].

The online tool predicted that *C. sinensis* 14-3-3 isoforms were localized in cytoplasm, chloroplast, nucleus plasma, and plasma membrane (Table 1), suggesting distinct and differential patterns of subcellular distribution. These isoforms exhibited a high cell-type specificity. The specificity of cellular and subcellular localization may contribute to their diverse interactions with targets as well as differential functions in cellular activities. *Arabidopsis* 14-3-3/GFP fusions experiment indicated that 14-3-3 localization is both isoform specific and highly dependent upon interaction with cellular clients [45]. Additionally, this interaction can alter the subcellular localization of target proteins [46]. In soybean, SGF14 proteins can regulate the nuclear-cytoplasmic movement of GmMYB176, which can alter the expression of *CHS8*, a gene in isoflavonoid biosynthesis [47]. 

### 3.2. CitGF14s Were Differentially Expressed in Various Organs of Citrus

The tissue-specific pattern of gene expression can provide important clues about gene function [48]. The expression of *CitGF14s* in different organs (Figure 4) may suggest that they are involved in various aspects of physiological and developmental processes. *CitGF14a*, *e*, *h*, and *i* were highly upregulated in different organs based on RNA-Seq raw data (Figure 4A). RT-qPCR analysis also showed the highly upregulation of *CitGF14a*, *c*, *d*, *h*, and *i* in shoots and *CitGF14c*, *e*, and *h* in leaves, which were principally similar to those from the RNA-Seq analysis. A discrepancy occurred in *CitGF14d* between RNA-Seq (Figure 4A) and RT-qPCR (Figure 4B) data. RNA-Seq data showed its down regulation in almost all tested tissue and organs, but it was dramatically unregulated in flower and also in shoot in RT-qPCR analysis. Such a disagreement could be attributed to the differences in cultivars and plant growth conditions, which needs further investigation. Nevertheless, the high level of *CitGF14d* transcript in flowers and shoot in contrast to the minimal levels in the other organs (Figure 4B) may suggest the specificity of *CitGF14d* in flower and leaf development. A similar expression pattern also occurred in another woody plant mulberry tree [49] where some *MaGF14s* were specifically expressed in certain organs. Additionally, soybean *14-3-3* isoforms also showed ubiquitous expression in all tissues, and different expression of *SGF14* genes in embryos during seed development indicated that *14-3-3s* may be involved in soybean seed development [36]. In cotton, Northern blotting and RT-qPCR analysis showed that *Gh14-3-3* genes were developmentally regulated in fiber development [50,51]. In an early report, a high level of *Arabidopsis 14-3-3ω* mRNA occurred in flowers. On the other hand, *14-3-3κ* and *14-3-3λ* expression did not show much difference across tissues [52]. The expression of different *CitGF14s* in different organs or in the same organs of citrus may indicate the versality of *CitGF14* involvement in citrus growth and development, which deserve further investigation. 

### 3.3. CitGF14s Were Induced by Citrus Canker and Greening Infections

Citrus canker and citrus greening are two notorious bacterial pathogens and have significantly affected citrus production worldwide. In this study, both microarray and RT-qPCR data clearly showed that *CitGF14s* are involved in citrus responses to canker (Figure 5). Microarray data indicated that both *CitGF14g* and *CitGF14i* were highly upregulated upon the infection of both Xaa and Xac (Figure 5A). RT-qPCR analysis further confirmed the upregulation of both *CitGF14g* and *CitGF14i* (Figure 5B) and found that *CitGF14b*, *d*, and *f* were also involved in the later period of Xac infection. These results indicate that 14-3-3 proteins may involve in the regulation of citrus canker resistance as well as functional redundancy in stress tolerance. 

The responses of *CitGF14s* to citrus greening pathogen were not pronounced as those to citrus canker based on the microarray results (Figure 5A). There was a slight increase in the expressions of *CitGF14c* and *f* in leaves and *CitGF14e* in peel, whereas *CitGF14a*, *g*, and *i* were downregulated in leaves, stems or fruit peel. How such up or down regulations affects the pathogen development is unknown. Considering the severity of citrus greening in the citrus industry and the role of 14-3-3 in regulation of plant responses to biotic stresses, further research on *CitGF14s* in regulation of citrus response to greening is warranted. 

Pathogen infection triggered differential expressions of *14-3-3* genes has been documented in other plants. In tomato, at least 10 *14-3-3* genes are differentially expressed in response to fungal toxin fusicoccin [32]. The tomato 14-3-3 protein TFT7 can bind with MAPKKKα and SIMKK2, resulting in programmed cell death associated with immunity [53]. TFT4 is another member of tomato *14-3-3* proteins that can bind with the effector XopQ from *Xanthomonas euvesicatoria* (Xcv) to suppress the effector-triggered immunity [54]. 

### 3.4. CitGF14s Differentially Responded to Abiotic Stresses

Phytohormones play a central role in plant defense responses to environmental stress. RT-qPCR analysis showed that all *CitGF14* genes responded to JA, ABA, ETH, and SA applications by either down or up regulation at different times (Figure 6A). Such variable responses may suggest that 14-3-3s could function as a multiple regulator in plant hormone signaling. Four rice *GF14* genes were induced by benzothiadiazole, JA, ETH, and H_2_O_2_ during pathogen attach [26]. Quantification of the 20R/16R promoter-driven GUS expression in different transgenic potato plants revealed that *14-3-3* isoforms can be induced by various stimuli, such as ABA, SA, NaCl, and metal ions [55]. The *Arabidopsis* 14-3-3 λ isoform was reported to specifically bind the C-terminal domain of RPW8.2, resulting in the enhanced resistance to powdery mildew fungus via the SA signaling pathway [56]. In the present study, *CitGF14b* was highly downregulated, but *CitGF14d* was strongly upregulated by ABA treatment. This may suggest that citrus *14-3-3s* could link to ABA in mediation of different stress responses. A study with barley 14-3-3 proteins as baits in yeast two-hybrid (Y2H) library resulted in the identification of 132 new molecular targets, including three ABA signal transduction related proteins (AREB/ABF/ABI5-like proteins) [57]. 

High and low temperatures as well as wounding induced variable expressions of all nine *CitGF14s* (Figure 6B). *CitGF14a*, *d*, *e*, and *g* that were upregulated at high temperature became down regulated in low temperature treatment. The reverse is true for *CitGF14b*, *c*, and *f*. Only *CitGF14h* and *i* had both up and down regulations in high and low temperature treatments. These results may indicate that different isoforms of *CitGF14s* were involved in response to two opposite temperature stresses. A study with *Arabidopsis* showed that overexpression of *14-3-3ε* was ineffective in cold tolerance, but overexpression of both ω and ε produced more cold tolerant plants [58]. Wounding also induced all *CitGF14s* gene expression including the upregulation of *CitGF14a* and *i* but downregulation of *CitGF14d* at variable times. These results concurred with a report by Lapointe et al. [59] that *14-3-3* mRNA was upregulated in poplar plants after wounding treatment. 

*CitGF14s* exhibited similar expression patterns in response to both NaCl and PEG-simulated drought stresses (Figure 6C). All *CitGF14s* genes were highly upregulated except *CitGF14d* that was downregulated, suggesting that all *CitGF14s* participated in citrus responses to NaCl and drought. These results are consistent with 14-3-3 responses to salinity and drought stresses in other plants [3,22,60]. An *Arabidopsis GF14λ* was introduced into cotton plants, resulting in improved drought tolerance with a “stay-green” phenotype. The stomata of the transgenic plants might be regulated by *GF14λ* through some transporters, such as H+-ATPase whose activities are controlled by their interaction with 14-3-3 proteins. In the present study, we noticed that *CitGF14d*, which was highly induced by ABA signal (Figure 6A), were downregulated under salinity and drought stresses (Figure 6C). This result may indicate that *CitGF14d* acted as a negative regulator. The overexpression of *GsGF14o* from *Glycine soja* in *Arabidopsis* resulted in down-regulation of a drought-responsive marker gene, the transgenic line showed reduction of stomatal development under drought treatment. Thus, the *Glycine soja* 14-3-3 gene *GsGF14o* functioned as a negative regulator of drought tolerance [61]. 

### 3.5. Prediction of Interactions among CitGF14 Proteins

Different genes with similar expression patterns, commonly known as co-expressed genes were believed to be functionally related [62]. To improve our understanding of *CitGF14s*’ functions in *C. sinensis*, *CitGF14s* were integrated into the Network Inference for Citrus Co-Expression (NICCE) (http://citrus.adelaide.edu.au/nicce/home.aspx). A detailed *14-3-3* gene information and tabs were presented in Additional file 3: Table S2, which contained top 100 expressed genes in *C. sinensis* based on HRR (highest reciprocal ranks). HRR highlighted in black (bold), black, and grey colors signified statistical significance of HRR at *p* < 0.01, *p* < 0.05, and *p* > 0.05 levels, respectively. As described previously, the microarray data did not cover all the *CitGF14* genes, we only obtained co-expressed genes from six member of *CitGF14* genes in *C. sinensis* datasets. We found a number of genes co-expressed with three citrus canker responsible *CitGF14s* (*CitGF14g*, *CitGF14h*, and *CitGF14i*). They are leucine-rich repeat family proteins, zinc finger (C3HC4-type RING finger) family proteins, and zinc finger (DHHC type) family proteins, WD-40 repeat family proteins, lesion inducing protein-related, and peroxidase 63. These proteins are involved in a variety of functions ranging from signal transduction and transcription regulation to defense responses. For example, the leucine-rich repeat family proteins are associated with innate immunity in plants, serving as the first line of defense against pathogens. Our results may indicate that *CitGF14g*, *CitGF14h*, and *CitGF14i* are involved in regulation of defense response to the infection of citrus canker pathogens. 

The interaction among 14-3-3 isoforms or individual isoforms with other proteins is important for understanding the biological functions of 14-3-3s. Y2H assay is a powerful tool widely used for identifying novel protein–protein interaction [63]. In the present study, CitGF14i was found to be able to interact with CitGF14a, b, c, d, g, or h to form a heterodimer and interact with itself to form a homodimer in yeast (Figure 7). Interactions were also found in other 14-3-3 protein pairs, including CitGF14g with b or c and CitGF14d with a or c. Among the isoforms, CitGF14i appears to be the most active and important one due to its interaction with six isoforms and also with itself. It is known that 14-3-3 isoforms have different affinities to individual targets; thereby, there is a possibility that regulation of specific processes could be accomplished by single 14-3-3 isoforms [16]. In this study, *CitGF14i* was highly up-regulated in flower and leaves or shoots in general (Figure 4), strongly induced by the infection of Xac (Figure 5B), largely down-regulated in response to the application of growth regulators, and variably expressed in abiotic treatments (Figure 6). Intriguingly, *CitGF14i* is the only one localized in plasma membrane. Whether its subcellular location contributes to such active interactions is unknown. Although the function of all *CitGF14s* deserve further investigation, specific attention should be given to *CitGF14i* for its interactions with other proteins. Y2H screens complemented with 14-3-3 protein affinity purification and tandem mass spectrometry are another powerful tool for identifying protein interactions. This method identified five 14-3-3 isoforms in 7-day-old barley. Some of proteins were identified as 14-3-3 targets in both Y2H and affinity purification including 14-3-3 proteins themselves [64]. In order to uncover the 14-3-3 signaling pathway in healthy and disease, a high-throughput data in VisANT graphs (http://visant.bu.edu) was used to graph and validate 14-3-3 protein interactions [65]. 

**Figure 8:**
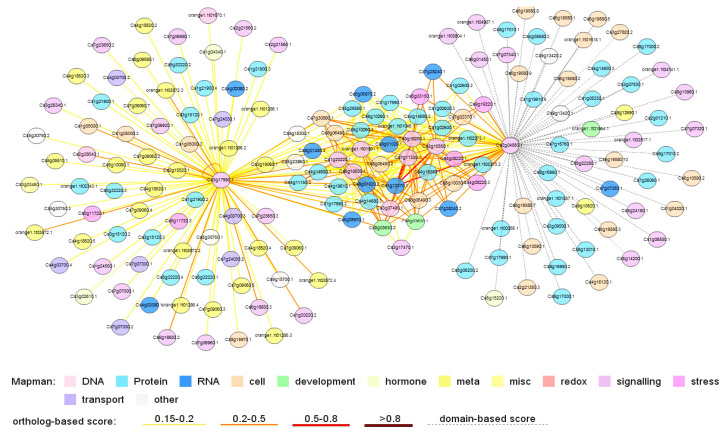
Protein–protein interaction network of CitGF14s and target proteins. The colored nodes represent proteins of miscellaneous functions in this network (see picture detail). Solid line or dotted line represents the predicted interaction based on ortholog or domain.

Two public citrus databases provided a genome-wide approach to predict 14-3-3 protein–protein interactions (PPI) or gene co-expression. Thus, a 14-3-3s protein interaction network was generated by CitrusNet (Figure 8). Among them, CitGF14a, e, and i play much greater roles than CitGF14g and h, which are more important than the remaining four CitGF14s. CitGF14d, CitGF14g, and CitGF14i contribute to their resistance to Xac infection, and CitGF14d could be a negative regulator in response to drought stress. With more than 150 nodes in the network, most nodes had different degrees of connection. Detailed connection among each CitGF14 and target was provided in Additional file 4: Table S3. More than 70 citrus 14-3-3 targets were identified, which participate in many molecular processes, including those involved in development, hormone, redox, signaling, stress, and transport. Polyubiquitin-A, clathrin heavy chain 1, heat shock protein 81-3, and heat shock protein 83 were protein nodes with the highest degree in CitrusNet [66], and these proteins also were found in 14-3-3 PPI network. Furthermore, casein kinase I, vesicle-fusing ATPase, serine/threonine-protein kinase TOR (TARGET OF RAPAMYCIN), histone deacetylase 6, leucine rich repeat-type serine/threonine receptor-like kinase were predicted to interact with several members of CitGF14s. TOR kinase is a client of CitGF14s. In *Arabidopsis*, TOR kinase was important in controlling plant growth, responding to environmental cues, and regulating cell processes [67]. In genome-wide PPI network, TOR kinase was a central part of citrus hormone cross-talk, which potentially interacted with proteins related to hormone signaling and hormone receptors [66]. Therefore, all these proteins play critical roles in CitGF14s regulating of various aspects of cellular processes. Furthermore, different members of CitGF14 interactions were found in both co-expression network and PPI network. Protein BRASSINOSTEROID INSENSITIVE 1 and leucine rich repeat-type serine/threonine receptor-like kinase appeared in PPI network, and they were also predicted as *14-3-3s* co-expression genes.

## 4. Materials and Methods

### 4.1. Identification of 14-3-3 Genes in Citrus sinensis

The *14-3-3* genes from *Arabidopsis* [6], soybean [36], and *Populus* [38] were employed as queries to perform a local BLASTP against the database: Orange Genome Annotation Project (http://citrus.hzau.edu.cn/orange/), which were retrieved from previous studies and database respectively [40]. Proteins with e-value belonging to a significant match (e-value < 10^−5^) in the blast analysis were considered as potential 14-3-3 members. The resulting protein sequences were examined with Pfam (http://pfam.sanger.ac.uk/search) and SMART (http://smart.embl-heidelberg.de/) to ensure the presence of 14-3-3-specific domains.

### 4.2. Chromosome Location of 14-3-3 Genes and Their Protein Properties and Sequence Analyses

The chromosome locations of *14-3-3s* genes were searched in the database of Orange Genome Annotation Project (http://citrus.hzau.edu.cn/cgi-bin/gb2/gbrowse/orange/) [43]. The molecular weight (MW) and isoelectric point (PI) of non-redundant genes were calculated by the online tool ExPASy (http://www.expasy.org/tools/). Subcellular localization was performed using WoLF PSORT at the website http://www.genscript.com/wolf-psort.html. All 14-3-3 amino acid sequences were used for identifying three-dimensional structure of 14-3-3 proteins using the SWISS-MODEL (https://swissmodel.expasy.org). To identify cis-regulatory elements, the 1.5 kb upstream regions to the translation start codon were selected from the Orange Genome Annotation Project and analyzed with PlantCARE (http://bioinformatics.psb.ugent.be/webtools/plantcare/html/) databases. The Gene Structure Display Server (http://gsds.cbi.pku.edu.cn) [68] was used to display gene structure models. 

### 4.3. Sequence Alignment and Phylogenetic Analysis of 14-3-3 Proteins

The amino acid sequences of *14-3-3* genes from different plant species were aligned using the software Clustal_X (version 1.83) with default parameters [69]. The unrooted phylogenetic trees were constructed based on alignments using MEGA 4.0 with the neighbor-joining method [70]. The bootstrap test was carried out with 1000 replicates.

### 4.4. RNA-Seq and Microarray Data Analysis

After the identification and characterization of *14-3-3* genes in citrus, publicly available RNA-Seq raw data and microarray database were mined for *14-3-3* expression profiles in different organs of citrus and in response to abiotic and biotic stresses. 

The expression of *14-3-3s* in callus, leaf, flower, and fruit were analyzed using RNA-Seq raw data available in *C. sinensis* genome database (http://citrus.hzau.edu.cn/orange/). The expression level of *14-3-3* genes was calculated as Log2 based RPKM. The expression data were hierarchically clustered with average linkage and displayed in HemI [71].

Affymetrix microarray data obtained from the NCBI Gene Expression Omnibus (GEO) database under the series accession number GSE33003, GSE33004, and GSE10798 were mined for citrus *14-3-3* response to biotic stresses. In experiments with Ca. Las infection (GSE33003 and GSE33004), young, healthy Valencia sweet orange plants were graft-inoculated with budwood from Ca. Las-infected citrus plants. The leaf, stem, and root samples were collected from three symptomatic and three healthy control trees for RNA extraction and analyzed using microarrays [72,73]. For citrus canker (Xac) or (Xaa) infection (GSE10798), adult leaves of sweet orange were infiltrated with the bacterial suspensions or water (mock control). Leaves samples were collected after bacterial infiltration for RNA extraction and hybridization on Affymetrix microarrays [74]. The microarray CEL files were normalized using Robust Multi-array Average (RMA) in R/Bioconductor (ver 2.15), and normalized data was used for identifying differential expressed genes [75,76]. The heatmap for the *14-3-3* transcripts with their expression values were performed by HemI [66].

### 4.5. Plant Materials and Treatments

To confirm the expression of *14-3-3* genes in different organs of *C. sinensis*, root, stem, leaf, and shoot samples were taken from one-year old seedling of *C. sinensis*. Flower, fruit peel, juice, and seed samples were collected from eight-year-old adult trees. Four biological samples per organ were frozen in liquid N and stored in −80 °C. 

Further analysis of *14-3-3* genes in response to various stresses were conducted using *C. sinensis* “Xuegan”. Seeds of “Xuegan” were germinated in a plant growth chamber at 25 °C under a photoperiod of 16-h light/8-h dark with a relative humidity of 70%. The light was provided by fluorescent white-light tubes. At the two-leaf stage, seedlings were transplanted singly into pots (15 × 15 cm) filled with Fafard Professional Potting Mix (Sun Gro Horticulture, Agawam, MA, USA). The following treatments were applied to the plants, each experiment was arranged as a complete randomized design with four replicate per treatment. 

Infection with citrus canker: The bacterial strain Xac 29-1 was cultured in NA nutrient broth with 1.5% agar at 28 °C for 36 h. Cultured bacterial cells were washed twice with sterile water and then resuspended in sterile water to a final concentration OD600 = 0.3. The diluted cells were infiltrated into leaves with a needleless syringe, sterile water was injected into leaves as the control treatment. Leaf samples were collected after 0, 2, 6, 12, 48, 96, and 192 h of inoculation, respectively. 

Hormone treatments: Seedlings were sprayed with 1 mM SA, 100 µM JA, 100 µM ABA, and 100 µM ETH solutions, respectively. Leaf samples were collected after 0, 2, 6, 12, 24, and 48 h of the application, respectively. Leaf samples collected from seedlings sprayed with distilled water at the corresponding time were considered the control treatment. 

Low or high temperature and wounding treatments: Seedlings were incubated in a growth chamber with temperature of 4 °C or 42 °C for 48 h. For wounding treatment, each fully expanded leaf was penetrated with a needleless syringe 10 times. Leaf samples were collected at 0, 2, 6, 12, and 48 h, respectively. Seedlings grown in the chamber with the temperature at 25 °C as the control, and leaf samples were collected at the same times as those of treated seedlings. 

Salt and drought treatments: Seedlings were grown in the potting mix were drenched with 200 mM NaCl or 20% PEG6000 solution until leachate appeared from the bottom of pots (about 250 mL solution was used for each treatment). Seedlings were irrigated with 250 mL water as the control treatment. Leaf samples were collected from treated and control seedlings after 0, 2, 6, 12, 48, 96, and 144 h of treatment, respectively.

All the collected leaf samples were immediately frozen in liquid N and stored at −80 °C for RNA extraction.

### 4.6. RNA Isolation and Expression Analysis

Total RNA was extracted from the collected samples using the RNAprep pure Plant Kit (Tiangen, Beijing, China) according to the manufacturer’s instructions. One thousand nanograms of total RNA was used to synthesize first-strand cDNA with EasyScript One-Step gDNA Removal and cDNA Synthesis SuperMix (Transgen, Beijing, China). Quantitative PCR was carried out using TransStart Tip Green qPCR SuperMix (Transgen, Beijing, China) on a CFX96 Real-time System (Bio-Rad, Hercules, CA, USA) according to the manufacturer’s protocol. *GAPDH* [77], *Actin*, and *F-box* [78] were used as reference genes to normalize the expression of the investigated genes. Gene specific primers (Additional file 5: Table S4) were designed with the Primer Premier 6 software (Premier Biosoft International, San Francisco, CA, USA). The PCR reaction mixtures were incubated at 95 °C for 30 s, followed by 40 cycles of 95 °C for 5 s and 60 °C for 30 s. The relative expression levels were determined by ^2−ΔΔ^Ct method described by Livak and Schmittgen [79], data were analyzed using SPSS 22.0 (IBM Corporation, Somers, NY, USA) statistics software, and mean differences were separated by Tukey’s HSD test at *p* < 0.05 level with four biological replicates. The heatmap for the *14-3-3* transcripts with their expression values were generated using HemI tool [66].

### 4.7. Prediction of Cis-Regulatory Elements

The sequences of 1.5 kb upstream regions from the translation initiation codon of each gene of 14-3-3s were selected and subjected to analysis by the online database PlantCare. Putative developmental and stress-responsive cis-elements in citrus *14-3-3s* were identified.

### 4.8. Gene Co-Expression Network and Protein–Protein Interaction Prediction

To predict gene interaction on a genome-wide scale, keyword 14-3-3s were searched in publicly accessible tool NICCE (http://citrus.adelaide.edu.au/nicce/home.aspx) to predict potential targets of 14-3-3s [80]. CitrusNet and PPI networks in *C. sinensis* were constructed using ortholog-based and domain-based interaction methods, which contained 8195 proteins and 124,491 interactions [65]. The nine citrus 14-3-3 proteins were used as hub nodes to connect potential target proteins in CitrusNet (http://citrus.hzau.edu.cn/orange/ppi/index.php). All 14-3-3s and client proteins were linked into an interconnected sub-network which was visualized by Cytoscape. 

### 4.9. Analysis of CitGRFs Interactions by Y2H Assay

The full-length coding sequences of 9 citrus 14-3-3 proteins were introduced into the pGBKT7 fusion bait vector and pGADT7 fusion prey vector. The fused pGADT7-CitGF14s and pGBT9-CitGF14s recombinant vectors were then co-transformed into yeast strain Y2H gold by LiAc/SS carrier DNA/PEG method [81]. The transformants were first selected in the SD/-Trp-Leu medium and PCR testing. After that the positive colonies were transferred to the selection medium supplemented with X-α-gal but lacked Trp, Leu, His, and adenine (SD/-Trp-Leu-His-Ade). Aureobasidin A (AbA) was added to the selection plates to suppress the auto-activation of the prey vectors. 

## 5. Conclusions

The present study identified nine *14-3-3 genes* (*CitGF14a* through *CitGF14i*) in *C. sinensis* through genome-wide analysis. All the *CitGF14s* genes were detected in different tissues or organs but varied in abundance. Transcript levels of *CitGF14s* were also analyzed after plants were treated with hormones, extreme temperatures, drought, salinity, wounding, and infected with Xac 29-1 strains. Almost all *CitGF14s* responded to the treatments by variable levels of expression during the experiments, suggesting that *CitGF14s* play important roles in citrus responses to different exogenous and endogenous signals. This study also showed that most gene family members had a functional divergence of 14-3-3 proteins. Y2H assay showed that CitGF14i was the most active and important isoform due to its interaction with six other isoforms and also with itself. Additionally, CitGF14d, CitGF14g, and CitGF14i contribute to their resistance to Xac infection, and CitGF14d could be a negative regulator in response to drought stress. Finally, a citrus 14-3-3 interactome network was constructed by PPI method and microarray gene co-expression. Our study for the first time provides a comprehensive framework about *14-3-3* family genes in *C. sinensis*, which may lead to further investigation of their roles in citrus growth and development as well as in response to abiotic and biotic stresses.

## Figures and Tables

**Table 1:** Identified nine *CitGF14* (*14-3-3*) genes from the whole genome of *Citrus sinensis*.

Name	Gene ID	*Arabidopsis* Orthologue	Chr. No.	Chr. Location	ORF (bp)	Length (aa)	PI	MW (kDa)	Subcellular Localization
*CitGF14a*	Cs2g04850.1	AT2G42590.1	Chr 2	2,529,656–2,533,444	774	258	4.69	29.433	Cyto
*CitGF14b*	Cs2g15550.4	AT2G42590.2	Chr 2	12,364,752–12,368,242	798	266	4.72	29.951	Chlo
*CitGF14c*	Cs3g18200.1	AT1G34760.1	Chr 3	21,756,849–21,761,115	759	253	4.92	28.861	Cyto
*CitGF14d*	Cs7g11330.1	AT1G26480.1	Chr 7	7,462,893–7,465,189	795	265	5.14	30.227	Cyto
*CitGF14e*	Cs1g20220.2	AT5G65430.2	Chr 1	23,332,994–23,337,303	741	247	4.83	27.946	Nucl_plas
*CitGF14f*	Cs3g17470.1	AT5G65430.1	Chr 3	21,178,807–21,181,787	756	252	4.76	28.536	Nucl_plas
*CitGF14g*	Cs3g17990.1	AT1G78300.1	Chr 3	21,561,464–21,564,313	795	265	4.69	29.742	Nucl_plas
*CitGF14h*	Cs6g18830.1	AT5G38480.1	Chr 6	18,853,688–18,857,047	789	263	4.75	29.740	Nucl_plas
*CitGF14i*	Or1.1t01991.1	AT1G78300.1	chrUn	31,516,661–31,519,401	783	261	4.84	29.442	Plas

## Data Availability

The data presented in this study are available in article and supplementary materials.

## Supplementary Materials

The following are available online at https://www.mdpi.com/1422-0067/22/2/568/s1, Additional file 1: Figure S1. *CitGF14s* sequence alignment and protein homology modeling; Additional file 2: Table S1. *Cis*-acting regulatory elements; Additional file 3: Table S2. 14-3-3s Co-expressed genes. Additional file 4: Table S3. Domain-based 14-3-3s PPI network; Additional file 5: Table S4. Gene specific primers.
