# Supplementary material for: Molecular Analysis of 14-3-3 Genes in Citrus sinensis and Their Responses to Different Stresses
Source: Int J Mol Sci. 2021 Jan 8;22(2):568. doi: 10.3390/ijms22020568 (PMC7826509; doi:10.3390/ijms22020568)
Supplement: Supplementary file 1 [file ijms-22-00568-s001.zip › Figure S1. CitGF14s sequence alignment and protein homology modeling.docx]

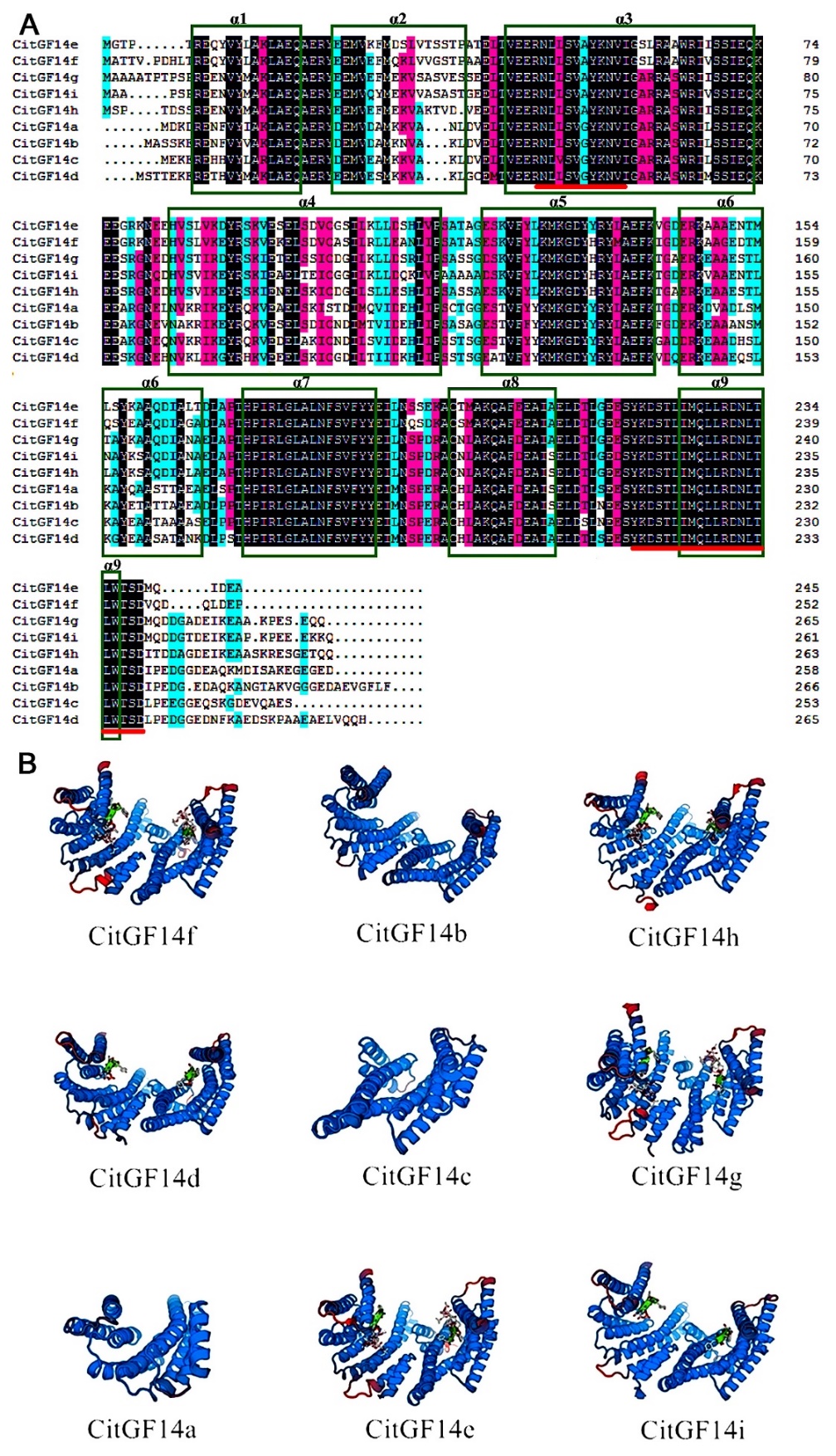


**Figure S1.** *CitGF14s* sequence alignment and protein homology modeling. **A**. Amino acid sequence alignment among CitGF14s. The blue shaded letters represented identical amino acids, and less similar amino acids are shaded pink. The α-helices were marked in green rectangular boxes. **B**. 14-3-3 protein homology modeling. These predicted models represent diverse three dimensional 14-3-3 protein conformations.
