# Supplementary material for: Molecular Analysis of 14-3-3 Genes in Citrus sinensis and Their Responses to Different Stresses
Source: Int J Mol Sci. 2021 Jan 8;22(2):568. doi: 10.3390/ijms22020568 (PMC7826509; doi:10.3390/ijms22020568)
Supplement: Supplementary file 1 [file ijms-22-00568-s001.zip › Table S1. Cis-acting regulatory elements.docx]

**Table S1.** *Cis*-acting regulatory elements presented in the promoters of *14-3-3* genes in *Citrus sinensis.*

| **Gene** | **Motifs related to growth and development** | **Motifs related to stress response** |
| --- | --- | --- |
| **CitGF14a** | A-box, circadian | ARE, CGTCA-motif, GC-motif, HSE, MBS, TC-rich repeats, TGACG-motif |
| **CitGF14b** | Skn-1_motif, circadian | ABRE, ARE, GARE-motif, HSE, MBS, P-box |
| **CitGF14c** | MSA-like, NON-box, Skn-1_motif, circadian | ABRE, ARE, Box-W1, CGTCA-motif, HSE, LTR, MBS, TC-rich repeats, TGACG-motif |
| **CitGF14d** | A-box, CCGTCC-box,  O2-site, RY-element | ARE, Box-W1, HSE, MBS, SARE, TCA-element |
| **CitGF14e** | CAT-box, GCN4_motif, O2-site, Skn-1_motif, circadian | ABRE, ARE, Box-W1, HSE, MBS, TC-rich repeats, TGA-element |
| **CitGF14f** | GCN4_motif, O2-site, Skn-1_motif, circadian | ARE, AuxRR-core, HSE, LTR, MBS, TC-rich repeats, TCA-element, wound-responsive element |
| **CitGF14g** | AACA_motif, O2-site, Skn-1_motif, circadian | ABRE, ARE, CGTCA-motif, ERE, GC-motif, HSE, MBS, P-box, TC-rich repeats, TCA-element, TGA-element, TGACG-motif |
| **CitGF14h** | circadian | Box-W1, GARE-motif, GC-motif, HSE, MBS, P-box, TATC-box, AT-rich sequence |
| **CitGF14i** | Skn-1_motif, circadian | ARE, Box-W1, EIRE, LTR, MBS, TC-rich repeats, TCA-element, WUN-motif |
